# Increased probability of mood disorders after age-related macular degeneration: a population-based cohort study

**DOI:** 10.1038/s41598-022-19429-5

**Published:** 2022-09-08

**Authors:** Chia-Yi Lee, Hung-Chi Chen, Jing-Yang Huang, Chi-Chun Lai, Hung-Yu Lin, Shun-Fa Yang, Wei-Chi Wu

**Affiliations:** 1 Institute of Medicine, Chung Shan Medical University, No. 110, Sec. 1, Chien-Kuo N. Rd., Taichung, 40201 Taiwan; 2 Nobel Eye Institute, Taipei, Taiwan; 3 Department of Ophthalmology, Jen-Ai Hospital Dali Branch, Taichung, Taiwan; 4 Department of Ophthalmology, Chang Gung Memorial Hospital, 5 Fuxing Street, Guishan District, Taoyuan, Taiwan; 5 Department of Medicine, Chang Gung University College of Medicine, Taoyuan, Taiwan; 6 Center for Tissue Engineering, Chang Gung Memorial Hospital, Linkou, Taiwan; 7 Department of Medical Research, Chung Shan Medical University Hospital, Taichung, Taiwan; 8 Department of Ophthalmology, Chang Gung Memorial Hospital, Keelung, Taiwan; 9 Department of Ophthalmology, Show Chwan Memorial Hospital, Changhua, Taiwan; 10 Department of Optometry, Chung Shan Medical University, Taichung, Taiwan

**Keywords:** Macular degeneration, Depression, Epidemiology

## Abstract

We aim to investigate the association of mood disorders with age-related macular degeneration (AMD). This retrospective cohort study used data from 2000 and 2016 from National Health Insurance Research Database (NHIRD) in Taiwan. Patients with AMD diagnosis formed the exposed group, and an age- and sex-matched group without AMD served as the nonexposed group. Main outcomes were the incidence of mood disorders including psychological counseling, behavior therapy, sleep or anxiety-related disorders, and major depressive disorders (MDDs) in the exposed and non-exposed groups. The Cox proportional hazard regression analysis was used to evaluate the incidence and adjusted hazard ratio (aHR) of mood disorders. A total of 5916 and 11,832 individuals with and without AMD were enrolled into the exposed and nonexposed groups. There were 1017 (17.19%) and 1366 (11.54%) episodes of mood disorders occurred in the exposed and nonexposed groups, respectively. The aHRs of any psychological counseling, behavioral therapy, sleep or anxiety-related disorders, and MDD were significantly higher in patients with AMD than in those without AMD (all *P* < 0.05). Besides, patients with dry-AMD, participants aged 50–70 years, and women with AMD had a higher incidence of mood disorders (all *P* < 0.05) than did non-AMD individuals, patients > 70 years, and women without AMD. In conclusion, AMD occurrence leads to an increased rate of mood disorders, particularly among those with dry-AMD, middle aged participants (aged 50–70), and women.

## Introduction

Age-related macular degeneration (AMD) is the leading cause of legal blindness worldwide^1^. In East Asia, AMD prevalence is estimated to be > 11% in the population > 75 years old^2^. According to clinical manifestation, advanced AMD comprises two subtypes: dry AMD (dry-AMD) and neovascular AMD (nAMD; characterized by choroidal neovascularization)^1^. AMD treatment is decided based on the AMD subtype, where anti-vascular endothelial growth factor (anti-VEGF) therapy is widely applied for wet-AMD management, whereas no effective treatment is available for dry-AMD, although some treatments have been proposed^1,3–5^.

Severe visual impairment resulting from AMD could lead to difficulty in performing daily activities^6^. In patients with prominent manifestations of AMD receiving medical treatment, fall risk is significantly increased compared with non-AMD individuals of similar age^7,8^. In addition, the risk of hip bone fracture is high in the AMD population, with an increased rate of total hip replacement^9^. Conversely, cognitive function decreases in patients with AMD, particularly in those with dual sensory loss^10^. Accordingly, AMD could influence the psychological status in such populations due to impairment in daily functions and social abilities. Severe eye diseases may be associated with affective disorder, with a prevalence rate of 11–57% in patients with glaucoma and 16–42% in patients with AMD^11–13^.

Several studies have proposed a relationship between AMD and mood disorders, including major depression disorder (MDD), because of decreased visual function^6,14–16^. A previous study, using the Geriatric Depression Scale Short Form, showed that depression episodes were more in patients with AMD than in general patients^17^, and another population-based study demonstrated the relationship between depressive disorder and nAMD^14^. The National Eye Institute Visual Function Questionnaire and its variants have been used to evaluate the effectiveness of management protocols for depressive disorder in patients with AMD^18–20^. Depressive disorder frequently occurs in patients with AMD and severe visual impairment^21,22^, and anxiety and depression may occur in patients with nAMD undergoing anti-VEGF therapy^23^. The impact of age and sex on mood disorders in patients with AMD requires further investigation.

The current study evaluated the correlation between AMD and subsequent mood disorders using data from the National Health Insurance Research Database (NHIRD) in Taiwan. The effects of different AMD subtypes, age, and sex on mood disorder incidence were analyzed.

## Result

**Figure 1:**
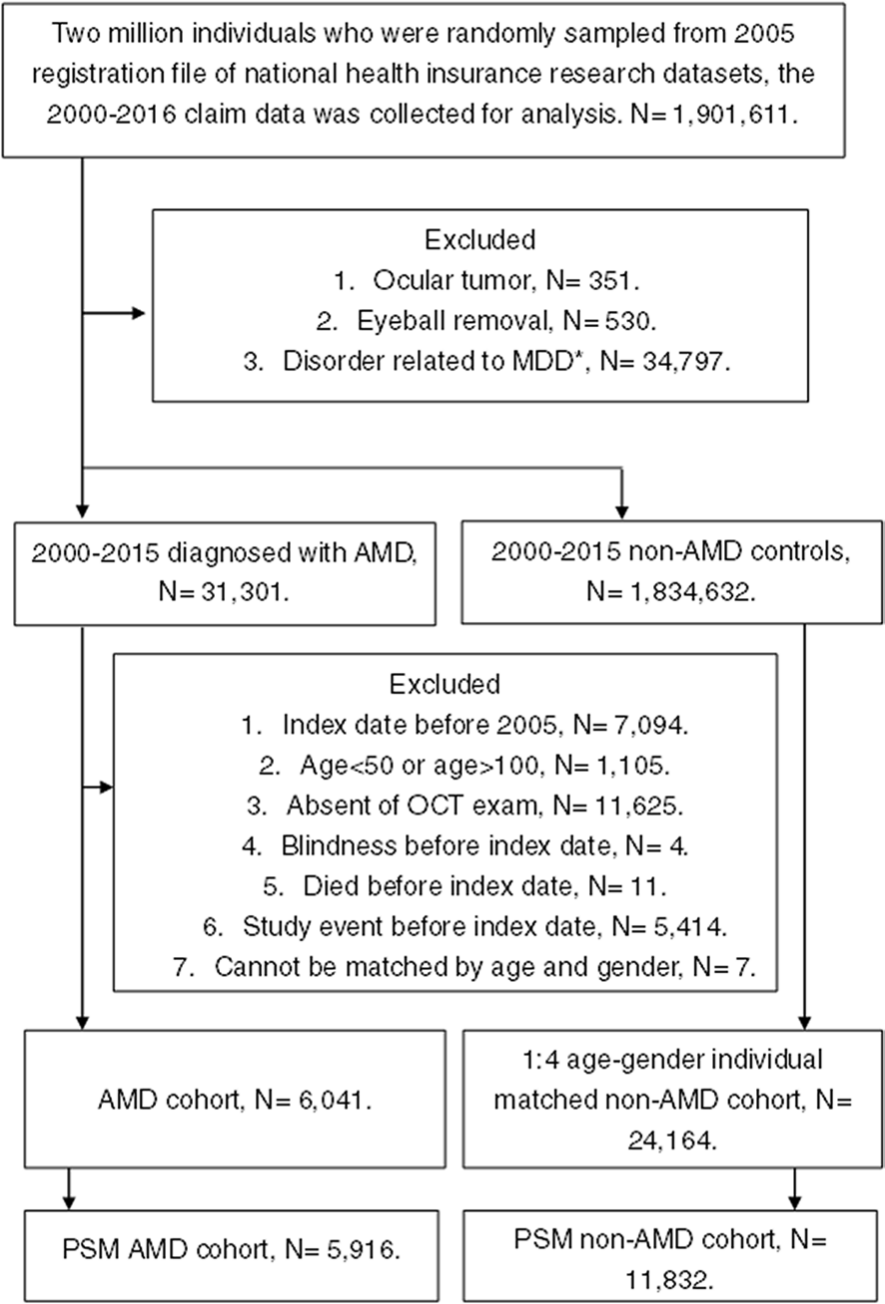
Flowchart of patient selection. *AMD* age-related macular degeneration, *MDD* major depression disorder. *Including schizophrenia, bipolar disease, chronic pain, personality disorders, physical and sexual abuse, sexual and gender identity disorders, eating disorders, and post-traumatic stress disorder.

A total of 5916 patients diagnosed as having AMD were enrolled into the exposed group, and 11,832 non-AMD individuals were enrolled into the nonexposed group. The flowchart of patient selection as well as exclusion is presented in Fig. 1. Due to propensity score matching, no significant difference was observed between the two groups in terms of age, sex, education level, marital status, and systemic comorbidities, except for hypertension (*P* = 0.0157) (Table 1). Regarding treatment application, 1320 patients in the exposed group who received anti-VEGF therapy were considered to have a diagnosis of nAMD (Table 1).

**Table 1:** Basic characteristics of the study and control groups.

Basic characteristics	Study	Control	*P*
N	5916	11,832	
**Age (years)**			0.1859
50–70	3473 (58.71%)	6823 (57.67%)	
71–100	2443 (41.29%)	5009 (42.33%)	
**Sex**			0.4916
Male	3441 (58.16%)	6818 (57.62%)	
Female	2475 (41.84%)	5014 (42.38%)	
**Education (years)**			0.3370
< 6	2965 (50.12%)	6088 (51.45%)	
6–9	822 (13.89%)	1607 (13.58%)	
9–12	1574 (26.61%)	3094 (26.15%)	
≥ 12	555 (9.38%)	1043 (8.82%)	
**Marital status**			0.3114
Unmarried	240 (4.06%)	437 (3.69%)	
Married	4826 (81.58%)	9739 (82.31%)	
Divorced	282 (4.77%)	509 (4.3%)	
Widowed	568 (9.60%)	1147 (9.69%)	
**Comorbidities**			
Hypertension	2675 (45.22%)	5577 (47.13%)	0.0157*
Diabetes mellitus	1604 (27.11%)	3246 (27.43%)	0.6508
Ischemic heart diseases	632 (10.68%)	1285 (10.86%)	0.7195
Hyperlipidemia	1390 (23.50%)	2787 (23.55%)	0.9302
Congestive heart failure	211 (3.57%)	405 (3.42%)	0.6220
Peripheral vascular disease	120 (2.03%)	217 (1.83%)	0.3711
Cerebrovascular disease	463 (7.83%)	896 (7.57%)	0.5493
Dementia	50 (0.85%)	79 (0.67%)	0.1895
Chronic pulmonary disease	518 (8.76%)	1056 (8.92%)	0.7088
Rheumatic disease	51 (0.86%)	94 (0.79%)	0.6371
Peptic ulcer disease	537 (9.08%)	1079 (9.12%)	0.9265
Kidney disease	366 (6.19%)	699 (5.91%)	0.4608
Liver disease	465 (7.86%)	921 (7.78%)	0.8587
Hemiplegia or paraplegia	29 (0.49%)	56 (0.47%)	0.8778
Malignancy	366 (6.19%)	734 (6.20%)	0.9649
Alcohol-related disorders	3 (0.05%)	4 (0.03%)	0.5929
Drug-related disorders	15 (0.25%)	30 (0.25%)	1.0000
**AMD subtype**			
Dry-AMD	4596 (77.69%)		
nAMD	1320 (22.31%)		

*N* number, *AMD* age-related macular degeneration, *nAMD* neovascular age-related macular degeneration.

*Significant difference.

**Figure 2:**
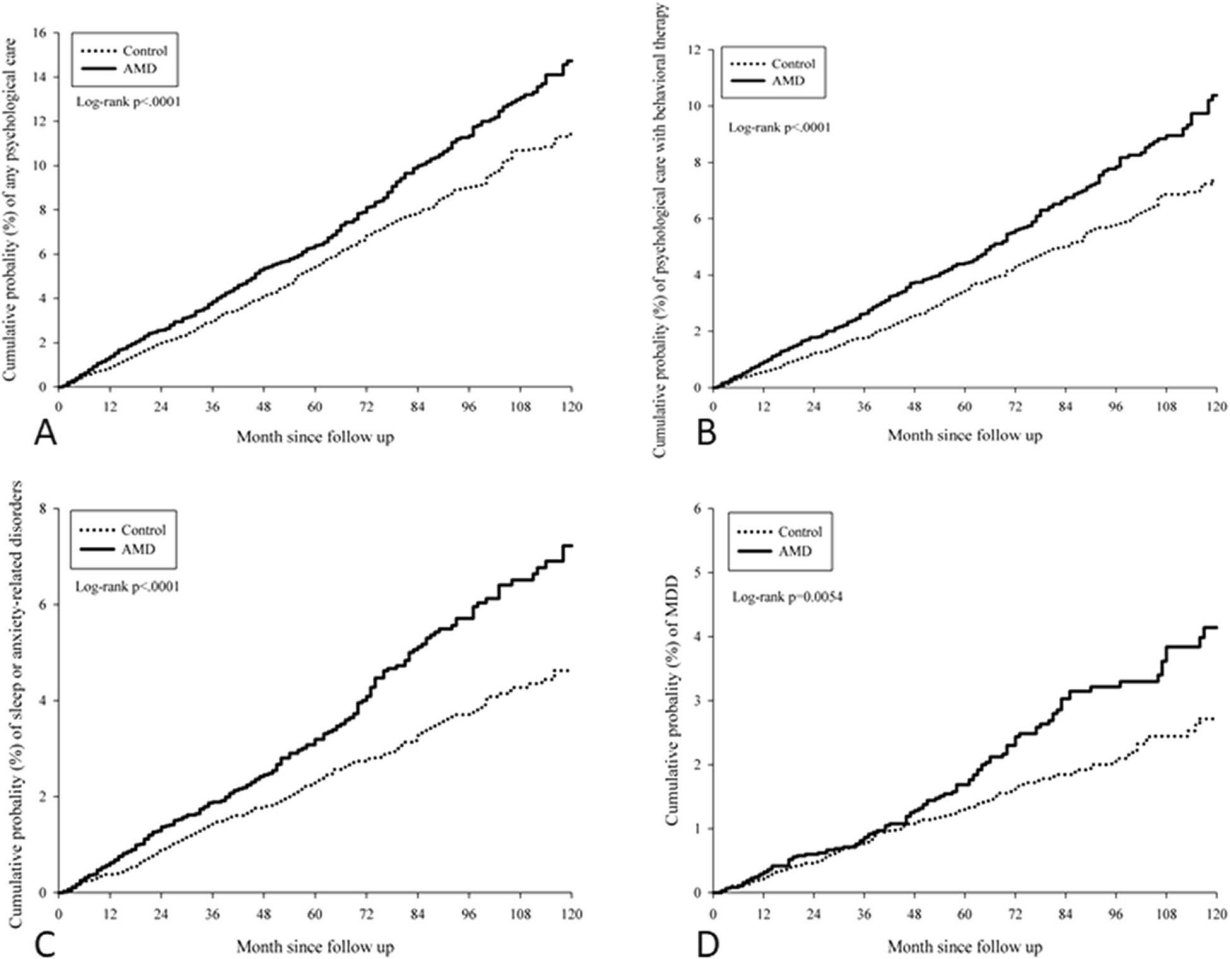
Cumulative probabilities of the mood disorders between the study and control groups. (**A**) Cumulative probability of psychiatric outpatient visit. (**B**) Cumulative probability of behavior therapy. (**C**) Cumulative probability of sleep and anxiety disorder. (**D**) Cumulative probability of major depression disorder. *AMD* age-related macular degeneration.

During the follow-up period of up to 16 years, 1017 (17.19%) and 1366 (11.54%) events of mood disorders were reported in the exposed and nonexposed groups, respectively. The crude incidence rates of all mood disorders were higher in the exposed group than in the nonexposed group. Moreover, the aHRs of psychological disorder (aHR 1.26, 95% CI 1.11–1.43, log-rank *P* < 0.0001), behavioral therapy requirement in psychiatric OPD (aHR 1.37, 95% CI 1.17–1.60, log-rank *P* < 0.0001), sleep or anxiety-related disorders (aHR 1.47, 95% CI 1.22–1.77, log-rank *P* < 0.0001), and MDD (aHR 1.40, 95% CI 1.09–1.79, log-rank *P* = 0.0054) were significantly higher in patients with AMD than in those without AMD after adjustment for multiple potential risk factors (Table 2). In addition, the cumulative probability indicated a significantly increased incidence of all mood disorders in patients with AMD over time (log-rank *P* < 0.05; Fig. 2).

**Table 2:** Study events in the study and control groups.

Event	AMD group			Control group			aHR (95% CI)
	Person-months	Event	Rate^#^	Person-months	Event	Rate*	
Any psychological care	331,912	418	12.59 (11.44–13.86)	614,305	596	9.70 (8.95–10.51)	1.26* (1.11–1.43)
Behavioral therapy	336,789	286	8.49 (7.56–9.54)	621,096	376	6.05 (5.47–6.70)	1.37* (1.17–1.60)
Sleep or anxiety-related disorders	339,238	201	5.92 (5.16–6.80)	624,832	250	4.00 (3.53–4.53)	1.47* (1.22–1.77)
MDD	343,792	112	3.26 (2.71–3.92)	629,644	144	2.29 (1.94–2.69)	1.40* (1.09–1.79)

*AMD* age-related macular degeneration, *aHR* adjusted hazard ratio adjusted for age, sex, education, marital status, and comorbidities, *CI* confidential interval, *MDD* major depression disorder.

*Significant difference.

^#^Crude incidence rate per 10,000 person months.

In the subgroup analysis, individuals diagnosed with dry-AMD demonstrated a significantly higher risk of any psychological disorder (aHR 1.29, 95% CI 1.12–1.47, log-rank *P* = 0.0004), behavioral therapy requirement in psychiatric OPD (aHR 1.43, 95% CI 1.21–1.68, log-rank *P* < 0.0001), sleep or anxiety-related disorders (aHR 1.60, 95% CI 1.32–1.95, log-rank *P* < 0.0001), and MDD (aHR 1.38, 95% CI 1.06–1.80, log-rank *P* = 0.0510) than those without AMD (Table 3). Subsequent subgroup analyses indicated that those aged 50–70 years with AMD had a higher probability of any psychological disorder (aHR 1.51, 95% CI 1.26–1.80, log-rank *P* < 0.0001), requiring behavioral therapy in psychiatric OPD (aHR 1.60, 95% CI 1.29–1.98, log-rank *P* < 0.0001), sleep or anxiety-related disorders (aHR 1.59, 95% CI 1.24–2.03, log-rank *P* < 0.0001), and MDD (aHR 1.60, 95% CI 1.13–2.25, log-rank *P* = 0.0036) than those aged 50–70 years without AMD (Table 4). Significant interactions between age and AMD were observed in the frequency of receiving any psychological care (*P* = 0.002) and behavioral therapy (*P* = 0.02).

**Table 3:** Study events in different types and treatments of age-related macular degeneration and control groups.

Event	Rate (per 1000 person months)			aHR1 (95% CI)	aHR2 (95% CI)
	Non-AMD, n = 11,832	Dry-AMD, n = 4596	nAMD, n = 1320		
Any psychological care	0.97 (0.90–1.05)	1.29 (1.16–1.43)	1.16 (0.94–1.44)	1.285 (1.123–1.470)	1.177 (0.940–1.476)
Behavioral therapy	0.61 (0.55–0.67)	0.89 (0.78–1.01)	0.71 (0.54–0.92)	1.427 (1.211–1.681)	1.157 (0.869–1.540)
Sleep or anxiety-related disorders	0.40 (0.35–0.45)	0.65 (0.56–0.76)	0.40 (0.28–0.57)	1.602 (1.318–1.947)	1.006 (0.692–1.463)
MDD	0.23 (0.19–0.27)	0.32 (0.26–0.4)	0.34 (0.23–0.49)	1.380 (1.056–1.803)	1.461 (0.961–2.221)

*AMD* age-related macular degeneration, *nAMD* neovascular age-related macular degeneration, *n* number, *aHR* adjusted hazard ratio adjusted for age, sex, education, marital status, and comorbidities, *CI* confidential interval, *MDD* major depression disorder, *aHR1* the hazard ratio of dry-AMD compared with non-AMD individuals, *aHR2* the hazard ratio of nAMD compared with non-AMD individuals.

*Significant difference.

^#^Crude incidence rate per 10,000 person months.

**Table 4:** Age stratified hazard ratio for mood disorders in patients with AMD compared with individuals without AMD.

Event	Incidence rate^#^ (95% CI)		aHR (95% CI)
	Non-AMD	AMD	
**Any psychological care**			P for interaction = 0.002
Age 50–70 years	7.36 (6.55–8.28)	11.38 (9.99–12.95)	1.51 (1.26–1.80)*
Age 71–100 years	13.53 (12.12–15.11)	14.45 (12.53–16.65)	1.01 (0.84–1.21)
**Behavioral therapy**			P for interaction = 0.020
Age 50–70 years	4.70 (4.06–5.44)	7.72 (6.60–9.02)	1.60 (1.29–1.98)*
Age 71–100 years	8.26 (7.18–9.51)	9.67 (8.14–11.49)	1.11 (0.89–1.39)
**Sleep or anxiety-related disorders**			P for interaction = 0.346
Age 50–70 years	3.55 (3.00–4.20)	5.71 (4.77–6.85)	1.59 (1.24–2.03)*
Age 71–100 years	4.73 (3.93–5.69)	6.25 (5.05–7.74)	1.29 (0.97–1.72)
**MDD**			P for interaction = 0.205
Age 50–70 years	1.80 (1.42–2.28)	2.98 (2.33–3.83)	1.60 (1.13–2.25)*
Age 71–100 years	3.07 (2.45–3.86)	3.68 (2.79–4.85)	1.16 (0.81–1.66)

*AMD* age-related macular degeneration, *aHR* adjusted hazard ratio adjusted for age, sex, education, marital status, and comorbidities, *CI* confidential interval, *MDD* major depression disorder.

*Significant result for null hypothesis of aHR = 1.

^#^Crude incidence rate per 10,000 person-months.

Furthermore, women with AMD had a significantly higher risk of any psychological disorder (aHR 1.54, 95% CI 1.27–1.86, log-rank *P* < 0.0001), behavioral therapy requirement in psychiatric OPD (aHR 1.68, 95% CI 1.33–2.13, log-rank *P* < 0.0001), sleep or anxiety-related disorders (aHR 1.43, 95% CI 1.09–1.87, log-rank *P* = 0.069), and MDD (aHR 1.57, 95% CI 1.10–2.25, log-rank *P* = 0.0147) than women without AMD, whereas such a correlation was only found in men with AMD who had sleep or anxiety-related disorders (aHR 1.50, 95% CI 1.16–1.94, log-rank *P* = 0.0014) compared with men without AMD (Table 5). Significant interactions between sex and AMD were observed in the frequency of receiving any psychological care (*P* = 0.008) and behavioral therapy (*P* = 0.027).

**Table 5:** Sex stratified hazard ratio for mood disorders in patients with AMD compared with individuals without AMD.

Event	Incidence rate^#^ (95% CI)		aHR (95% CI)
	Non-AMD	AMD	
**Any psychological care**			P for interaction = 0.008
Male	10.16 (9.15–11.28)	11.45 (10.03–13.07)	1.09 (0.92–1.29)
Female	9.11 (8.03–10.33)	14.15 (12.32–16.26)	1.54 (1.27–1.86)*
**Behavioral therapy**			P for interaction = 0.027
Male	6.50 (5.71–7.40)	7.95 (6.79–9.31)	1.18 (0.96–1.45)
Female	5.48 (4.66–6.44)	9.23 (7.78–10.95)	1.68 (1.33–2.13)*
**Sleep or anxiety-related disorders**			P for interaction = 0.802
Male	3.62 (3.04–4.30)	5.50 (4.55–6.64)	1.50 (1.16–1.94)*
Female	4.50 (3.77–5.38)	6.50 (5.31–7.96)	1.43 (1.09–1.87)*
**MDD**			P for interaction = 0.400
Male	2.19 (1.75–2.73)	2.88 (2.22–3.74)	1.27 (0.90–1.78)
Female	2.41 (1.90–3.07)	3.76 (2.89–4.90)	1.57 (1.10–2.25)*

*AMD* age-related macular degeneration, *aHR* adjusted hazard ratio which adjusted for age, sex, education, marital status, and comorbidities, *CI* confidential interval, *MDD* major depression disorder.

*Significant result for null hypothesis of aHR = 1.

^#^Crude incidence rate per 10,000 person-months.

## Discussion

The results indicate that the probability of mood disorders is significantly higher in individuals diagnosed with AMD compared with individuals without AMD. This includes mood disorders requiring psychiatric OPD consultation or behavioral therapy, sleep or anxiety-related disorders, and MDD. In the subgroup analysis, patients with dry-AMD, patients aged 50–70 years with AMD, and women with AMD exhibited a higher incidence of mood disorders (all *P* < 0.05). These data may support the view that poor vision contributes to the psychological burden of patients with AMD, particularly in the aforementioned high-risk groups.

The impairment of visual performance, mainly visual acuity, affects the performance of daily activities and psychophysiological function substantially^16,24^. Because one of the prominent manifestations of AMD is the decrement of central vision^25^, motion disability was reported in patients with AMD^26^, and the quality of life would also be diminished in this population^27^. In addition to restrictions in daily activities, patient with AMD are susceptible to bone fracture and osteoarthritis, particularly in the older adults population^9^. In a previous study, the incidence of femur neck fracture was significantly higher in the AMD population than in the non-AMD population^9^. Furthermore, the rate of hip fracture was higher in patients with AMD and glaucoma than in the nonexposed group without visual impairment^28^. Severe visual loss affects physical and cognitive function^29^. In studies that have surveyed those with prominent dual sensory loss involving visual and hearing defects, cognitive function was significantly lower than in the population without sensory disorders^10,30^. Dementia disorder such as Alzheimer disease is strongly associated with AMD^31^, which may result from both the degenerative nature of the two diseases and the lack of visual stimulation, contributing to decreased cognitive function^1,32,33^.

Depressive disorders have been often found in those with severe visual impairment irrespective of their age^34–36^. Vision loss in patients with advanced AMD, diabetic retinopathy, and chronic uveitis has been found to be associated with increased rates of anxiety, sleep disorder, and depression^15,37,38^. Moreover, dementia, which is related to AMD, is correlated with depression^31,39^. Furthermore, depressive disorder can lead to dementia, and the use of antidepressants may reverse this course ^40^, and post-stroke depression is common, although the exactpathophysiology needs further validation^41^. Evidence suggests that mood disorders may be related to certain degenerative vascular or nervous damage, which is also observed in neurodegenerative disorders and AMD^31^. Consequently, AMD may be associated with mood disorders, and the effect may be vary in different populations. The findings of the current study may support such a correlation.

Some studies have proposed an association between AMD and mood disorders^6,14,15,17^. Popescu et al. found that patients with visual impairment had a higher risk of depressive disorder, particularly those with AMD^17^. Another population-based study conducted in East Asia suggested that the rate of depressive disorders was significantly high in the nAMD population^14^. Chung et al. did not consider the medical prescription for patient enrollment. In addition, they did not consider the coexisting medical condition, marital status, and education of the patients. The current study considered all these factors to increase the accuracy of the analysis because all these factors could affect psychological stress^42,43^.

The current study revealed that dry-AMD was significantly correlated with mood disorders. Conversely, a previous study demonstrated a significant aHR of depressive disorders in patients with nAMD^14^. However, the previous study only adjusted for the monthly income, geographical location, and urbanization level of the study population^14^, whereas the current study considered additional factors such as the basic health status and mood disorder-related comorbidities, including congestive heart failure and malignancy, in the multivariable analysis^44,45^. Furthermore, the current study excluded those with mild mood disorders, as indicated by psychiatric OPD visit before the index date, because individuals with mood distress have an increased possibility of developing severe conditions like MDD^43^. Considering the disease course and treatment of AMD, visual acuity in nAMD can be preserved if treated properly and promptly^3,4^, whereas effective therapy is lacking for dry-AMD with retinal atrophy^25^. The quality of life significantly increased after intravitreal aflibercept injection in patients with nAMD^3^, and a similar outcome was recorded in patients with nAMD who received ranibizumab therapy, with increased quality of life and ability to read newspaper^46^. As a consequence, the improved quality of life after treatment targeting nAMD may prevent mood disorders and subsequent depressive disorders among patients. Vision loss in those with advanced dry-AMD is more irreversible than those with nAMD. This may lead to severe and persistent psychological stress and subsequent mood disorders. Regarding AMD distribution, dry-AMD prevalence was approximately 77.69% in all individuals with AMD, which is slightly lower than that in a previous study^47^.

In the subgroup analysis stratified by age, the frequencies of mood disorder risks were higher in individuals aged 50–70 years with AMD compared with individuals without AMD aged 50–70 years. In the age-based subgroup analyses, however, the general incidence of mood disorders was higher in the 70–100-year-old population than in the 50–70-year-old population. Patients aged 50–70 years with AMD have an increased probability of developing mood disorders for various reasons compared with their counterparts without AMD. In a multicentered study, the prevalence of anxiety disorders was reported to be significantly higher in patients aged 65–69 years than in those aged 75–79 and 80–84 years^48^. We hypothesize that some patients aged 65–69 years would be working and would be under the financial burden of educating their children or paying their house loan. Consequently, the necessity of working and the early diagnosis of AMD (compared with their older counterparts) may cause tremendous psychological stress on individuals aged 50–70 years old with AMD; thus, their rate of mood disorders is universally higher than that of similarly aged individuals without AMD. With regards to patients with AMD aged 50–70 years and 70–100 years, the differences in receiving psychological care and behavioral therapy between those with and those without AMD were significantly greater in the 50–70-year-old group compared with the 70–100-year-old group. Therefore, the early development of AMD may indeed cause more distress compared with the relatively late development of AMD. In addition, patients who develop early AMD may experience higher rates of mood disorders compared with patients without AMD of a similar age. Further studies are still needed to prove these hypotheses.

Female patients with AMD exhibited higher incidence of all mood disorders compared with female patients without AMD. The differences in frequency of receiving mental health care or behavioral therapy between populations with and without AMD were significantly greater among women than among men. Women are more vulnerable than men to various psychiatric distresses^43,49^; moreover, women are more willing than men to seek for medical assistance, particularly for mental health concerns^50^. The study results indicate that women with AMD are at a higher risk of developing all types of mood disorders than are women without AMD. Perimenopausal status is associated with higher probability and severity of depressive disorder than premenopausal status^51^; this may result from the reduction of estrogen, altering hippocampal morphology and function^52^. This phenomenon may further explain the significantly higher rate of mood disorders in women than in men in the current study because the women included in the current study were all > 50 years and were under perimenopausal status. By contrast, men with AMD had an increased risk of only sleep or anxiety-related disorders compared with men without AMD. These results may imply that stress resulting from AMD-related visual loss is more prominent in women than in men, which needs additional investigation.

The prevalence of AMD has been high in previous epidemiological studies^1,2,25^. The prevalence of AMD is > 3.21% in the Chinese population aged > 49 years^2^, whereas the 15-year incidence of AMD was 29.5% in the Western population according to the Blue Mountains Eye Study^53^. Furthermore, mood disorders including depressive and anxiety disorders affect a major portion of the population, with a life-time prevalence of 16% for MDD and a life-time prevalence of 5.9% for generalized anxiety disorder^43,54^. The number of people with mood disorders could be underestimated because some patients might not visit a formal psychiatrist or they may opt for traditional management^55^. Because severe mood disorders may result in suicide and both AMD and mood disorders affect numerous people, we should evaluate the psychological condition of patients with prolonged AMD.

This study has some limitations. First, because of the retrospective design of the study, the general characteristics of the exposed and nonexposed groups were less homogenous despite the double-matching processes employed. The matching process itself led to a loss of study samples and may have impaired statistical power and external validity. The effects of attention-deficit/hyperactivity disorder, visual acuity, cataracts, and glaucoma were not considered in the current study; this may have affected the outcome evaluation. Because of the use of claims data rather than medical records, patients’ visual acuity, the exact maculopathy type (i.e., occult or classic choroidal neovascularization), the purpose of exam, the diagnosis details of mood disorders, and the therapeutic outcome for both AMD and mood disorders could not be assessed. Use of claims data also necessitated defining nAMD on the basis of records of anti-VEGF injection; this may have led to the underestimation of nAMD cases, especially of severe nAMD. The frequency of ophthalmic department visits may increase the accessibility of psychiatric care in the AMD population and contribute to access bias for mood disorders. High-risk populations with a history of mental health disorders were excluded in this study, and thus, the external validity is limited. In addition, the tracking system of our database stopped once the outcome occurred (the diagnostic code of mental disorder presented); thus, we could not determine whether the disease was short-term or long-term. Finally, a possibility of patients with dry-AMD receiving anti-VEGF therapy or patients with nAMD not receiving treatments still exists. Nevertheless, anti-VEGF therapy covered by the health insurance system needs to be approved by an ophthalmologist in terms of appropriateness of the treatment, making the possibility of such condition less likely.

In conclusion, AMD may be associated with mood disorders after adjustment for multiple confounders. Mood disorder risk was found to be higher in patients with dry-AMD, patients aged 50–70 years, and women. Because AMD treatment is a long-term procedure, it is expensive and may lead to vision loss; therefore, these patients might have high stress. Our findings may indicate that patients with advanced AMD and marked signs of psychological stress should be referred to a psychiatrist for the evaluation and management of mood disorders. Furthermore, large-scale clinical research is required to investigate early psychological intervention in those with AMD and the relationship between mood disorder prognosis in the AMD population and AMD severity.

## Materials and methods

### Ethic declaration and data source

This retrospective population-based cohort study was approved by both the National Health Insurance Administration and Institutional Review Board of Chung Shan Medical University (Project identification code: CS-17075). Besides, all methods in this study were performed in accordance with the Declaration of Helsinki in 1964 and its late amendments. Additionally, the need for informed consent was waived by these institutions. The NHIRD contains the insurance claims data of nearly all the inhabitants of Taiwan. In the current study, the claims data were obtained from the Longitudinal Health Insurance Database 2005 (LHID 2005), which contains the data of approximately 2 million patients. Between the NHIRD data intervals of January 1, 2000, and December 31, 2016, patients included in the LHID 2005 were randomly sampled from the 2005 NHIRD registry, and their data were linked. Both Ninth and Tenth Revisions of International Classification of Diseases were used for disease diagnosis in the database. The quality of the diagnosis is dependent on each physician who enters the diagnostic codes, and the system is refreshed approximately every 3 years to provide the latest medical data.

### Patient selection

Patients were defined as having AMD if their medical records indicated (1) an AMD diagnosis, (2) they had undergone optical coherence tomography or fundus photography before or on the date of AMD diagnosis, and (3) had received an AMD diagnosis and treatment by an ophthalmologist. Ophthalmologists diagnosed AMD on the basis of diagnostic signs including the presence of drusen, pigment abnormalities, focal atrophy, geographic atrophy, and choroidal neovascularization. These signs of AMD were observed using indirect ophthalmoscope, fundus photography, optical coherence tomography, fundus autofluorescence, fluorescein angiography, and indocyanine green angiography. The index date was the date of AMD diagnosis. The exclusion criteria for both groups were as follows: (1) receipt of a diagnosis of legal blindness before the index date; (2) receipt of a diagnosis of ocular tumors at any time; (3) receipt of any type of eyeball removal surgery at any time; and (4) receipt of a diagnosis of specific mood disorders that may be related to MDD, including schizophrenia, bipolar disease, chronic pain, personality disorders, sexual and gender identity disorders, eating disorders, posttraumatic stress disorder, and physical or sexual abuse before the index date. To accurately reveal the correlation between AMD and mood disorders, the following exclusion criteria were applied to exclude certain morbidities that could influence the result: (1) index date before 2005; (2) age < 50 or > 100 years; (3) absence of optical coherence tomography before the index date; (4) death before the index date; and (5) outcome occurrence before the index date. The reason for the exclusion of patients with an index date before 2005 is that patients with mood disorders may not have received medical care for a certain period, and thus, excluding patients with an index date between 2000 and 2005 provides us with a 5-year observation period to ensure the population with AMD did not have any mood disorders before the index date. In addition, each individual in the exposed group was age- and sex-matched with the nonexposed group, and participants in the exposed group who could not be age- and sex-matched to four non-AMD individuals were excluded. Subsequently, the exposed and nonexposed groups were matched again through propensity score matching according to demographic data and systemic diseases listed in the Demographic Variables and Comorbidities section. Similarly, participants in the exposed group who could not be propensity score-matched to individuals in the nonexposed group were excluded. Participants in the exposed group were categorized into different AMD subtypes including those who received anti-VEGF treatments (nAMD), such as ranibizumab, bevacizumab, or aflibercept, and those who did not receive active intravitreal injection (dry-AMD). The exposed and nonexposed groups were included at a 1:4 ratio after propensity score matching. The differences in the outcomes of these groups were analyzed.

### Main outcome measurement

The existence of mood disorders, which was the main outcome in the current study, was defined based on the following: (1) any psychological counseling, which was defined as a psychiatric outpatient department (OPD) visit; (2) arrangement of behavior therapy in psychiatric OPD; (3) receipt of a diagnosis of sleep or anxiety-related disorders with a relevant medication prescription; and (4) receipt of a diagnosis of MDD with a relevant medication prescription. To prevent outcome overestimation, the mood disorder outcome was considered to occur only in individuals who received introductory diagnostic codes related to mood disorders and associated medical prescriptions from a psychiatrist, and they were included in the current study. The diagnosis of each mood disorder category was determined by psychiatrists in clinics or hospitals. Diagnostic codes of mental health outcomes could only be entered by doctors in the psychiatric department of clinics and hospitals in Taiwan. Psychiatrists usually made the diagnosis on the basis of both diagnostic interviews and instruments in accordance with the criteria of the fourth and fifth editions of the *Diagnostic and Statistical Manual of Mental Disorders*, and we only evaluated the risk of new onset of mood disorder with a one-off assessment.

### Demographic variables and comorbidities

To ensure that the health statuses of the exposed and nonexposed groups were sufficiently comparable, we also evaluated the effect of age, sex, education level, marital status, and the following systemic comorbidities in a multivariate analysis model: hypertension, diabetes mellitus, ischemic heart diseases, hyperlipidemia, congestive heart failure, peripheral vascular disease, cerebrovascular disease, dementia, chronic pulmonary disease, rheumatic disease, peptic ulcer disease, kidney disease, liver disease, hemiplegia or paraplegia, malignancy, alcohol-related disorders, and drug-related disorders. We longitudinally tracked the data from the index date until the date of mood disorder diagnosis, withdrawal from the National Health Insurance program, or the end date of NHIRD, which is December 31, 2016. In addition, we did not use time-varying covariates to estimate the time-to-event risk of mood disorder in the current study.

### Statistical analysis

SAS version 9.4 (SAS Institute Inc, NC, USA) was used for all analyses. After propensity score matching of the exposed and nonexposed groups at a 1:4 ratio, the incidence rate and corresponding 95% confidence intervals (CIs) were calculated through Poisson regression. In the next step, multivariable analysis by using Cox proportional hazard regression was adopted to compute the adjusted hazard ratios (aHRs) through the incorporation of the aforementioned demographic data and systemic comorbidities. Subsequently, the aHRs for mood disorders of the dry-AMD subgroup and nonexposed group were analyzed. Additionally, we calculated the effect of AMD on mood disorder occurrence based on age and sex. The Kaplan–Meier curve was plotted to determine the cumulative incidence of mood disorders between the exposed and nonexposed groups, and the log-rank test was performed to compare the survival curves. Statistical significance was set at *P* < 0.05. A *P* value less than 0.0001 was depicted as *P* < 0.0001.

## Data Availability

The datasets generated and analyzed during the current study are not publicly available because the National Health Insurance Administration prohibits such action, but part of them are available from the corresponding author on reasonable request.

## Abbreviations

AMD: Age-related macular degeneration
nAMD: Neovascular age-related macular degeneration
MDD: Major depression disorder
NHIRD: National Health Insurance Research Database
LHID 2005: Longitudinal Health Insurance Database 2005 version
OPD: Outpatient department
CI: Confidence intervals
aHR: Adjusted hazard ratios
anti-VEGF: Anti-vascular endothelial growth factor
